# Outcomes of Patients Aged 75 Years or Older Undergoing Endotracheal Intubation: An 11-Year Single-Center Retrospective Study

**DOI:** 10.7759/cureus.113702

**Published:** 2026-07-31

**Authors:** Hideyuki Niwa, Katsuyuki Kojima, Miku Kuriki, Kazumi Hori, Koji Sakamoto, Ayako Nakayama, Makoto Ishii

**Affiliations:** 1 Department of Respiratory Medicine, Nagoya Ekisaikai Hospital, Nagoya, JPN; 2 Department of Respiratory Medicine, Tokai Central Hospital of the Mutual Aid Association of Public School Teachers, Gifu, JPN; 3 Department of Respiratory Medicine, National Hospital Organization (NHO) Nagoya Medical Center, Nagoya, JPN; 4 Department of Respiratory Medicine, Nagoya University Graduate School of Medicine, Nagoya, JPN; 5 Doctoral Program, Department of Integrated Health Sciences, Nagoya University Graduate School of Medicine, Nagoya, JPN

**Keywords:** extubation, geriatrics, mechanical ventilation, prognosis, tracheal intubation

## Abstract

Introduction

Older adults aged ≥75 years often experience poor outcomes after endotracheal intubation, yet evidence describing survival and successful extubation in this population remains limited. This study evaluated clinical outcomes and factors associated with survival to discharge and successful extubation among elderly patients requiring mechanical ventilation.

Methods

We conducted a retrospective study at Tokai Central Hospital, Japan, including patients aged ≥75 years who underwent endotracheal intubation between 2014 and 2024. Patients intubated for procedural or perioperative purposes were excluded. Clinical characteristics, comorbidities, residence status, dementia, post-cardiopulmonary resuscitation (CPR) status, albumin level, and underlying disease were extracted from electronic medical records. The primary outcome was survival to discharge, and the secondary outcome was successful extubation. Associations were analyzed using chi-square tests. This study was approved by the Institutional Review Board of Tokai Central Hospital (approval date: August 22, 2025; identifier: 2025081901).

Results

A total of 147 patients (median age, 82 years) were included. Survival to discharge was 31%, and 27% were successfully extubated. Poorer outcomes were observed in patients aged ≥80 years; those with post-CPR status, dementia, hypoalbuminemia, nursing home residence, or pulmonary disease; and were associated with lower survival and extubation rates. Cardiac disease was associated with higher survival (51%) and extubation rates (42%). The median survival time among patients who died during hospitalization was 11 days.

Conclusions

Among patients aged ≥75 years undergoing endotracheal intubation, both survival and extubation rates were low. Prognosis varied substantially by patient characteristics, with particularly poor outcomes in those with post-CPR status, dementia, or hypoalbuminemia. These findings highlight the need for individualized, goal-concordant decision-making when considering intubation in elderly patients.

## Introduction

With advances in medicine, the world is facing an unprecedentedly aging population. In Japan, according to estimates as of September 15, 2025, the population aged ≥65 years has reached a record high of 29.4% of the total population, whereas the population aged ≥75 years has reached 17.2% [1]. These statistics indicate that opportunities to treat patients aged ≥75 years are increasing in daily clinical practice. In Japan, individuals aged ≥75 years are classified as being in the “old-old” age group, and the healthcare system treats them differently from those aged <75 years [2].

Endotracheal intubation and mechanical ventilation are often necessary for the management of severe respiratory failure. However, the decision to intubate elderly patients requires careful consideration because of multimorbidity and frailty. In addition to medical considerations, the values and preferences of patients and their families regarding life-sustaining treatment, including ethical considerations, are also important. Elderly patients generally have poorer prognoses and lower rates of successful extubation than younger adults [3]. Intubated elderly patients are at high risk of death, and even if they survive, their quality of life may be significantly impaired [4].

The management of critically ill elderly patients, particularly in intensive care units, consumes substantial resources and increases healthcare costs [5,6]. Therefore, early decision-making regarding the risks and benefits of endotracheal intubation and mechanical ventilation in critically ill elderly patients is important.

Some studies have reported that more than 70% of elderly individuals prioritize quality of life over longevity [7]. In clinical practice, patients or their families often indicate that many elderly patients do not wish to undergo mechanical ventilation. As a result, data on the prognosis of elderly patients who undergo emergency endotracheal intubation remain limited, creating a barrier for clinicians when providing decision support.

Previous reports on patients aged ≥65 years receiving mechanical ventilation have shown that age ≥75 years is a risk factor for poor prognosis [3]. However, this does not necessarily mean that all patients aged ≥75 years should avoid endotracheal intubation. It is important to clarify which patients aged ≥75 years are most likely to benefit from mechanical ventilation with endotracheal intubation and which are not. Therefore, this study aimed to investigate the prognosis and likelihood of successful extubation among patients aged ≥75 years who underwent mechanical ventilation with endotracheal intubation at our hospital over an 11-year period and to identify the patient groups most likely to benefit from this treatment.

## Materials and methods

This retrospective study included patients aged ≥75 years who underwent endotracheal intubation and mechanical ventilation at Tokai Central Hospital (Gifu, Japan) between January 1, 2014, and December 31, 2024.

Patient selection

**Figure 1:**
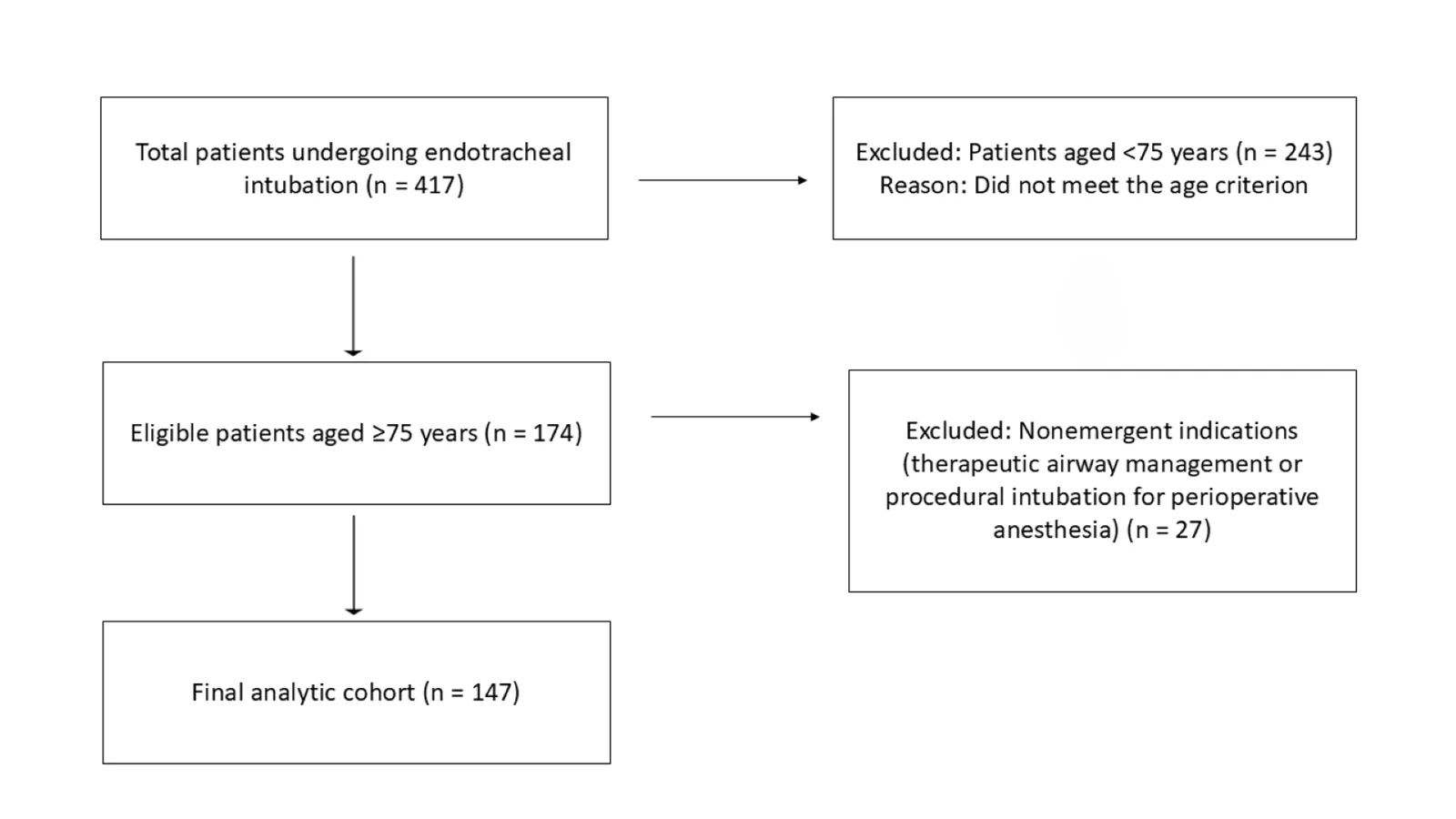
Patient selection.

All adult patients who underwent endotracheal intubation at Tokai Central Hospital between January 1, 2014, and December 31, 2024, were screened (n = 417). Among them, 243 patients younger than 75 years were excluded. The remaining 174 patients aged ≥75 years were eligible for evaluation. Of these, 27 patients were excluded because intubation was performed for nonemergent indications, including therapeutic airway management and procedural intubation for perioperative anesthesia. These cases did not represent acute medical deterioration requiring ventilatory support. Thus, the final analytic cohort consisted of 147 patients who underwent endotracheal intubation for acute medical conditions. The patient selection process is illustrated in Figure 1.

Data collection

Clinical data were extracted from electronic medical records using a standardized data collection form. Variables included age, sex, place of residence (home or nursing facility), indication for intubation, diagnosis at admission, and comorbidities [8-12]. All patients underwent oral endotracheal intubation, and the intubation method was determined by the attending physician.

Definitions

Airway disease was defined as conditions involving the upper airway, including choking, bronchial asthma, and foreign body aspiration; tongue-base obstruction secondary to other diseases was excluded. Lung disease was defined as disorders originating in the lung parenchyma, including aspiration pneumonia and other pulmonary infections, chronic obstructive pulmonary disease exacerbation, and pulmonary forms of acute respiratory distress; asthma exacerbation was not included in this category. Cardiovascular disease was defined as cardiac and circulatory conditions leading to respiratory failure, including heart failure, acute coronary syndromes, arrhythmias, and other hemodynamic disorders. Hypoalbuminemia was defined as a serum albumin level <2.0 g/dL at admission. Successful extubation was defined as remaining free from reintubation for ≥48 hours following extubation.

Outcomes

The primary outcome was survival to hospital discharge, and the secondary outcome was successful extubation.

Statistical analysis

Univariate analyses were performed using the chi-square test to evaluate associations between patient characteristics and outcomes. Multivariable logistic regression was not conducted because the sample size and the number of outcome events were insufficient to support a stable multivariable model, and the risk of overfitting was considered high. Given these limitations, the analyses were restricted to an exploratory univariate approach. ORs with 95% CIs were calculated using univariate logistic regression. Chi-square tests were used only to compare categorical variables and were not used to derive ORs. Statistical significance was defined as p < 0.05. Analyses were performed using IBM SPSS Statistics for Windows, version 30.0 (released 2024; IBM Corp., Armonk, NY, USA).

Ethical approval

This study was approved by the Institutional Review Board of Tokai Central Hospital (approval date: August 22, 2025; identifier: 2025081901). Patient confidentiality was maintained in accordance with institutional and ethical guidelines.

## Results

Patient characteristics

Between January 2014 and December 2024, 147 patients aged ≥75 years were enrolled in this study and underwent mechanical ventilation following endotracheal intubation. The patient characteristics are summarized in Table 1. The median age was 82 years (range, 75-90 years); 99 patients (67%) were aged ≥80 years, and 82 patients (55%) were male. Fifty-nine patients (40%) had post-cardiopulmonary resuscitation (CPR) status, 87 patients (59%) had dementia, 47 patients (32%) were nursing home residents, and 103 patients (70%) had diabetes. The underlying conditions included airway disease in 25 patients (17%), lung disease in 60 patients (41%), and cardiac disease in 53 patients (36%). In this study, 11 patients underwent tracheostomy.

**Table 1:** Patient characteristics. CPR, cardiopulmonary resuscitation

Characteristic	Value
Number of patients	147
Median age, years (range)	82 (75-90)
Age ≥80 years, n (%)	99 (67)
Sex, male/female	82/65
Post-CPR status, n (%)	59 (40)
Dementia, n (%)	87 (59)
Nursing home residence, n (%)	47 (32)
Diabetes, n (%)	103 (70)
Primary disease, n (%)	
Airway	25 (17)
Lung	60 (41)
Cardiovascular	53 (36)
Other	9 (6)

Primary outcome

Overall, 45 patients (31%) survived to hospital discharge. Among those who died during hospitalization, the median survival time was 11 days (range, 1-210 days). As shown in Table 2, survival to discharge differed across several subgroups. The 95% CIs are reported for each OR. Patients aged 75-79 years had a higher survival rate than those aged ≥80 years (42% vs 25%; OR, 2.1; 95% CI, 1.0-4.3; χ² = 4.1, df = 1, p = 0.043). Male patients had a higher survival rate than female patients (40% vs 18%; OR, 3.0; 95% CI, 1.4-6.3; χ² = 7.8, df = 1, p = 0.32). Post-CPR status was associated with lower survival (19%; OR, 0.36; 95% CI, 0.16-0.80; χ² = 6.2, df = 1, p = 0.013). Patients with dementia (13%; OR, 0.11; 95% CI, 0.05-0.24; χ² = 33, df = 1, p < 0.001), nursing home residents (9%; OR, 0.18; 95% CI, 0.07-0.43; χ² = 18, df = 1, p < 0.001), and those with hypoalbuminemia (10%; OR, 0.22; 95% CI, 0.05-0.98; χ² = 4.6, df = 1, p = 0.031) also had lower survival to discharge. The survival rate among patients with diabetes was 30% (OR, 0.79; 95% CI, 0.37-1.67; χ² = 0.36, df = 1, p = 0.55). When categorized by underlying disease, survival-to-discharge rates were 16% for airway disease (OR, 0.37; 95% CI, 0.11-1.18; χ² = 3.0, df = 1, p = 0.082), 20% for lung disease (OR, 0.41; 95% CI, 0.19-0.87; χ² = 5.4, df = 1, p = 0.020), and 51% for cardiovascular disease (OR, 4.4; 95% CI, 1.2-16; χ² = 5.2, df = 1, p = 0.023). Cardiovascular disease was associated with higher survival.

**Table 2:** Primary outcome. CPR, cardiopulmonary resuscitation

Predictor	Survival discharge				p-value	95% CI
	Yes (n = 45)	No (n = 102)	Survival rate (%)	OR		
Age 75-79 years, n (%)	20 (44)	28 (27)	42	2.1	0.043	1.0-4.3
Age ≥80 years, n (%)	25 (56)	74 (73)	25	-	-	-
Sex, male, n (%)	34 (76)	52 (51)	40	3.0	0.005	1.4-6.3
Sex, female, n (%)	11 (24)	50 (49)	18	-	-	-
Post-CPR status, n (%)	11 (24)	48 (47)	19	0.36	0.013	0.16-0.80
Dementia, n (%)	11 (24)	76 (75)	13	0.11	<0.001	0.05-0.24
Nursing home residence, n (%)	5 (11)	42 (41)	9	0.18	<0.001	0.07-0.43
Hypoalbuminemia, n (%)	2 (4.4)	18 (18)	10	0.22	0.031	0.05-0.98
Diabetes, n (%)	30 (67)	73 (72)	30	0.79	0.55	0.39-1.7
Primary disease						
Airway disease, n (%)	4 (8.9)	21 (21)	16	0.37	0.082	0.11-1.18
Lung disease, n (%)	12 (27)	48 (47)	20	0.41	0.02	0.19-0.87
Cardiovascular disease, n (%)	27 (60)	26 (25)	51	4.4	0.023	1.2-15
Other, n (%)	2 (4.4)	7 (7.0)	-	-	-	-

Secondary outcome

A total of 40 patients (27%) were successfully extubated. As shown in Table 3, extubation success also varied across several subgroups. ORs and 95% CIs are reported for each comparison. Patients aged 75-79 years had a higher extubation rate than those aged ≥80 years (33% vs 24%; OR, 1.6; 95% CI, 0.74-3.3; χ² = 1.3, df = 1, p = 0.25). Male patients had a higher extubation rate than female patients (31% vs 21%; OR, 2.0; 95% CI, 0.73-5.7; χ² = 1.8, df = 1, p = 0.18). Post-CPR status was associated with lower extubation success (14%; OR, 0.27; 95% CI, 0.11-0.63; χ² = 8.9, df = 1, p = 0.003). Patients with dementia had an extubation rate of 18% (OR, 0.34; 95% CI, 0.16-0.72; χ² = 8.3, df = 1, p = 0.004). Patients with hypoalbuminemia had an extubation rate of 5% (OR, 0.12; 95% CI, 0.02-0.92; χ² = 5.8, df = 1, p = 0.016). The extubation rate among patients with diabetes was 29% (OR, 1.4; 95% CI, 0.61-3.2; χ² = 0.65, df = 1, p = 0.42). When categorized by underlying disease, extubation rates were 20% for airway disease (OR, 0.62; 95% CI, 0.21-1.8; χ² = 0.80, df = 1, p = 0.37), 20% for lung disease (OR, 0.55; 95% CI, 0.27-1.1; χ² = 2.7, df = 1, p = 0.10), and 42% for cardiovascular disease (OR, 3.0; 95% CI, 1.5-6.2; χ² = 8.7, df = 1, p = 0.003). Cardiovascular disease was associated with higher extubation success.

**Table 3:** Secondary outcome. CPR, cardiopulmonary resuscitation

Predictor	Extubation				p-value	95% CI
	Yes (n = 40)	No (n = 107)	Extubation rate (%)	OR		
Age 75-79 years, n (%)	16 (40)	32 (30)	33	1.6	0.25	0.74-3.3
Age ≥80 years, n (%)	24 (60)	75 (70)	24	-	-	-
Sex, male, n (%)	27 (67)	59 (55)	31	2.0	0.18	0.73-5.7
Sex, female, n (%)	13 (33)	48 (45)	21	-	-	-
Post-CPR status, n (%)	8 (20)	51 (48)	14	0.27	0.003	0.11-0.63
Dementia, n (%)	16 (40)	71 (66)	18	0.34	0.004	0.16-0.72
Nursing home residence, n (%)	8 (20)	39 (36)	17	0.44	0.057	0.19-1.0
Hypoalbuminemia, n (%)	1 (2.5)	19 (20)	5	0.12	0.016	0.02-0.92
Diabetes, n (%)	30 (75)	73 (68)	29	1.4	0.42	0.61-3.2
Primary disease						
Airway disease, n (%)	5 (13)	20 (19)	20	0.62	0.37	0.21-1.8
Lung disease, n (%)	12 (30)	48 (45)	20	0.55	0.10	0.27-1.1
Cardiovascular disease, n (%)	22 (55)	31 (29)	42	3.0	0.003	1.5-6.2
Other, n (%)	1 (2.5)	8 (7.5)	-	-	-	-

## Discussion

When providing medical care to elderly patients, aggressive treatment, including endotracheal intubation, is not necessarily the optimal approach. These procedures not only impose a significant physical and psychological burden on patients and their families but may also lead to the overuse of medical resources [13]. Previous studies of patients aged ≥65 years who received mechanical ventilation have reported that no clear benefit from aggressive treatment was observed in patients aged ≥75 years [3]. Therefore, many elderly patients with severe underlying conditions do not wish to receive aggressive treatment. However, evidence sufficient to fully explain the benefits and risks of endotracheal intubation when requested by patients aged ≥75 years with severe respiratory failure remains limited.

This study examined outcomes following endotracheal intubation in patients aged ≥75 years, defined in Japan as the “old-old age” [2]. The primary outcome, the proportion of patients discharged alive, was 31%, and the secondary outcome, successful extubation, was 27%, indicating that fewer than one-third of the patients ultimately survived to discharge and that the overall benefit of intubation in this population was limited.

Factors associated with survival to discharge in this study included age 75-79 years, male sex, and the presence of cardiovascular disease. Conversely, factors associated with poorer outcomes included female sex, age ≥80 years, post-CPR status, dementia, hypoalbuminemia, residence in a nursing facility, and underlying lung disease. In particular, the significantly worse prognosis among patients aged ≥80 years, even within the ≥75-year age group, is noteworthy. Poor prognosis among ICU patients aged ≥85 years has been reported previously, suggesting that age may be an important prognostic factor even within the population of older adults aged ≥75 years [14].

Although the reasons for sex differences are unclear, the relatively favorable prognosis observed in patients with cardiovascular disease suggests that there may be a subgroup of older adults for whom appropriate therapeutic interventions can improve outcomes [15]. Conversely, the poor prognosis among patients with lung disease observed in this study is consistent with previous studies reporting poor outcomes associated with lung disease in the elderly population [16,17]. Dementia and institutionalization have previously been reported as factors associated with poor prognosis, and similar results were obtained in this study [3,9]. Furthermore, the poor prognosis of elderly patients following CPR is consistent with the findings of previous studies [18]. These findings are consistent with multiple reports indicating poor prognosis in critically ill elderly patients with multiple comorbidities [19-21].

Cardiovascular disease was associated with favorable outcomes, whereas post-CPR status, dementia, and hypoalbuminemia were associated with difficult extubation. The fact that differences were observed between survival-to-discharge and extubation outcomes suggests that tracheostomy may have had certain beneficial effects, even in cases where extubation was difficult. However, studies of patients with severe respiratory failure have reported that tracheostomy is a poor prognostic factor in elderly patients, suggesting that it does not consistently confer benefits in this population [22].

Taken together, these findings indicate that the prognosis of elderly patients aged ≥75 years who undergo endotracheal intubation is strongly influenced by preexisting conditions, functional status, and the underlying cause of respiratory failure. In particular, patients aged ≥80 years, those with dementia, and those with hypoalbuminemia had markedly poor outcomes, suggesting that careful consideration is required when discussing the appropriateness of aggressive treatment in these groups.

This study has several limitations. First, it was conducted at a single center, and the retrospective design may introduce selection bias, particularly regarding decisions to intubate. Second, several clinically important variables, including illness severity scores (Sequential Organ Failure Assessment (SOFA) Score, Acute Physiology and Chronic Health Evaluation II (APACHE II), and Simplified Acute Physiology Score (SAPS) II) and frailty assessments such as the Clinical Frailty Scale, were not collected, limiting our ability to adjust for baseline severity. Third, only univariate analyses were performed, and the absence of multivariable adjustment restricts the ability to account for potential confounding. These limitations may affect the interpretation of the findings.

Despite these limitations, this study provides clinically relevant information for physicians, patients, and families when considering endotracheal intubation in elderly individuals aged ≥75 years. The results highlight the importance of individualized decision-making based on age, comorbidities, functional status, and the underlying disease, rather than relying solely on chronological age.

## Conclusions

In this study of patients aged ≥75 years who underwent endotracheal intubation, fewer than one-third survived to hospital discharge, and only 27% were successfully extubated. Advanced age (particularly ≥80 years), dementia, hypoalbuminemia, post-CPR status, and underlying lung disease were associated with markedly poor outcomes, whereas cardiovascular disease was associated with comparatively favorable results. These findings highlight the substantial influence of preexisting conditions and functional status on prognosis in this population.

When considering endotracheal intubation for elderly patients aged ≥75 years, especially those with multiple comorbidities or frailty, clinicians should engage in careful, individualized decision-making that incorporates expected outcomes, patient values, and goals of care. Further prospective studies are needed to better define which subgroups of older adults may benefit from aggressive respiratory support and to guide shared decision-making for this growing population.
